# *Neoniphon pencei*, a new species of holocentrid (Teleostei: Beryciformes) from Rarotonga, Cook Islands

**DOI:** 10.3897/BDJ.3.e4180

**Published:** 2015-01-26

**Authors:** Joshua M. Copus, Richard L. Pyle, John L. Earle

**Affiliations:** † Hawaii Institute of Marine Biology, Kaneohe, United States of America; ‡ Bishop Museum, Honolulu, United States of America

**Keywords:** Holocentridae, *Neoniphon*, new species, Mesophotic Coral Ecosystems, MCE, Rarotonga, Cook Islands

## Abstract

*Neoniphon pencei*, n. sp., is described from thirteen specimens, 132-197 mm standard length (SL) collected from mesophotic coral ecosystems (MCEs) at Rarotonga, Cook Islands by divers using mixed-gas closed-circuit rebreathers. It differs from all other species of the genus in number of lateral line scales, scales above and below lateral line, elements of life color, and in COI and cytochrome b DNA sequences. Of the five other known species of *Neoniphon*, it is most similar to the Indo-Pacific *N. aurolineatus* and the western Atlantic *N. marianus* both morphologically and genetically.

## Introduction

Collections of shore fishes at mesophotic depths (~30-200 m) across the Indo-Pacific are yielding a surprising number of undescribed species (Pyle 2000). Here, we describe a new species of the genus *Neoniphon* from depths of 90-115 m at Rarotonga, Cook Islands, raising to six the number of species within this genus.

The genus *Neoniphon* Castelnau 1875 includes five currently recognized species: *N. argenteus* (Valenciennes in Cuvier and Valenciennes 1831), *N. aurolineatus* (Liénard 1839), *N. opercularis* (Valenciennes in Cuvier and Valenciennes 1831) and *N. sammara* (Forsskål 1775) from the tropical Indo-Pacific, and *N. marianus* (Cuvier in Cuvier and Valenciennes 1829) from the tropical western Atlantic. Woods and Sonoda (1973) placed these five species (referring to *N. argenteus* by the junior synonym *laevis* [=*læve*] Günther 1859, and to *N. aurolineatus* by the junior synonym *scythrops* Jordan and Evermann 1903) within the genus *Flammeo* Jordan and Evermann 1898, on the grounds that the earlier name *Neoniphon* (type species: *N. armatus* Castelnau 1875) was "based on a species whose status is uncertain" (p. 345). Randall and Heemstra (1985) treated the four Indo-Pacific species of the genus, and confidently asserted that the original description of *N. armatus* Castelnau 1875 (the type species of*Neoniphon*) is conspecific with *N. sammara*, and therefore considered *Neoniphon* as a valid genus with higher nomenclatural priority, to apply to the the four Indo-Pacific species as well as *N. marianus* from the tropical Atlantic. The lead author is working on a larger phylogeographic study involving this genus, which will be published at a later time.

## Materials and methods

Type specimens of the new species *Neoniphon pencei* from Rarotonga, Cook Islands have been deposited in the Bernice P. Bishop Museum, Honolulu (BPBM); the California Academy of Sciences, San Francisco (CAS); and the U.S. National Museum of Natural History, Washington, D.C. (USNM).

Measurements and counts given here follow the methods outlined in Randall (1998). Lengths of specimens are given as ratios of: standard length (SL) measured from the tip of the snout to the base of the caudal fin at the end of the hypural plate; body depth, taken at the point of maximum depth; or head length, measured from the median anterior point of the upper lip to the end of the longest opercular spine. Meristics and measurements were compared with data obtained from the literature for all five currently recognized species (Shimizu and Yamakawa 1979).

Tissue samples were obtained from each of the thirteen individuals of *N. pencei* collected at Rarotonga, Cook Islands by spear at 90-115 m. Tissue samples were also obtained from twenty-two specimens of the five other species of *Neoniphon*: *N. sammara* (n=6) collected from Diego Garcia, British Indian Ocean Territory; *N. opercularis* (n=2) collected from Moorea, French Polynesia; *N. aurolineatus* (n=7) collected from Oahu, Hawaii; *N. marianus* (n=1) collected from the Commonwealth of the Bahamas; and *N. argenteus* (n=6) collected from the Republic of Kiritimati. Total genomic DNA was extracted from each sample using the 'HotSHOT' protocol (Meeker et al. 2007). A 577-bp fragment of the mtDNA cytochrome *b* (*Cyt b>*) region was amplified using modified primers from Song et al. (1998) (5’-TGAAGTTGTCGGGATCTCCT-3’) and Taberlet et al. (1992) (5’-TGCCGTGACGTAAACTATGG-3’). Polymerase chain reaction (PCR) was performed in a 15 µl reaction containing 7.5 µl BioMix Red (Biolone Inc., Springfield, NJ, USA), 0.2 µM of each primer, 5-50 ng template DNA, and nanopure water (Thermo Scientific* Barnstead, Dubuque, IA, USA) to volume. PCR cycling parameters were as follows: initial 95°C denaturation for 10 min. followed by 35 cycles of 94°C for 30 sec, 60°C for 30 sec, and 72°C for 30 sec, followed by a final extension of 72°C for 10 min. PCR products were visualized using a 1.5% agarose gel with GelStarTM (Cambrex Bio Science Rockland, Inc., Rockland MA, USA) and then cleaned by incubating with 0.75 units of Exonuclease and 0.5 units of Shrimp Akaline Phosphate (ExoSAP; USB, Cleveland, OH, USA) per 7.5 µl of PCR product for 30 min. at 37°C followed by 85°C for 15 min. Sequencing was conducted in the forward direction and reverse direction when needed using a genetic analyzer (ABI 3130XL, Applied Biosystems, Foster City, California) at the Hawai'i Institute of Marine Biology EPSCoR Sequencing Facility. The sequences were aligned, edited and trimmed to a common length using Geneious Pro (v.5.6.6) DNA analysis software (Drummond et al. 2012). Twelve representative *Cyt b>* haplotypes were deposited in GenBank (accession numbers KJ188431-188436 and KJ201921-201926). jModelTest v.2.1.4 (Darriba et al. 2012, Guindon and Gascuel 2003) was used with an Akaike information criterion (AIC) test to determine the best nucleotide substitution model for the data. The GTR+G model with gamma parameter 0.1840 was identified to be the best suited model for phylogenetic inference. Maximum Likelihood, Neighbor-Joining, and Maximum Parsimony tree-building methods were implemented using Mega v.5.2.2 (Tamura et al. 2011). *Sargocentron rubrum* (Genbank accession number AP004432.1) was used to root a maximum likelihood phylogenetic reconstruction. Clade support was evaluated by bootstrapping 1,000 replicates in all cases (Felsenstein 1985).

A DNA barcode (cytochrome c oxidase I; COI) was completed for the holotype and one paratype (BPBM **XXXXX**) using the primers from Baldwin et al. (2009), Fish-BCH (5'-ACTTCYGGGTGRCCRAARAATCA-3') and Fish-BCL (5'-TCAACYAATCAYAAAGATATYGGCAC-3') using the following PCR protocol: initial 95°C denaturation for 10 min. followed by 35 cycles of 94°C for 30 sec, 55°C for 30 sec, and 72°C for 30 sec, followed by a final extension of 72°C for 10 min. All other procedures were as described above. Both individuals possessed the same COI haplotype, so only one record was deposited in GenBank (http://www.ncbi.nlm.nih.gov/; accession number KJ188437) and BOLD (www.boldsystems.org; dx.doi.org/10.5883/DS-NPE511).

## Taxon treatments

### Neoniphon pencei

Copus, Pyle, and Earle sp. n.

urn:lsid:zoobank.org:act:43F5CABA-6E4B-42BB-8569-8F93D3502DE9

NPE001-14

KJ201926

KJ188437

#### Materials

- **Type status:** Holotype. **Occurrence:** catalogNumber: 41197; recordedBy: David F. Pence; individualCount: 1; lifeStage: adult; preparations: 55% Isopropyl; disposition: in collection; associatedSequences: GenBank KJ201926 (*cyt b*); KJ188437 (COI); **Taxon:** taxonID: 43f5caba-6e4b-42bb-8569-8f93d3502de9; scientificNameID: 43f5caba-6e4b-42bb-8569-8f93d3502de9; acceptedNameUsageID: 43f5caba-6e4b-42bb-8569-8f93d3502de9; parentNameUsageID: b047f156-f8da-4ec6-9f64-87345b68a759; originalNameUsageID: 43f5caba-6e4b-42bb-8569-8f93d3502de9; nameAccordingToID: bbdce765-389b-4338-9c36-68def122f4fc; namePublishedInID: bbdce765-389b-4338-9c36-68def122f4fc; scientificName: Neoniphon pencei; acceptedNameUsage: *Neoniphon pencei* Copus, Pyle and Earle, 2014; parentNameUsage: Neoniphon Castelnau, 1875; originalNameUsage: *Neoniphon pencei* Copus, Pyle and Earle, 2014; nameAccordingTo: Copus, Joshua M., Richard L. Pyle & John L. Earle. 2014. *Neoniphon pencei*, a new species of holocentrid from Rarotonga, Cook Islands. Biodiversity Data Journal.; namePublishedIn: Copus, Joshua M., Richard L. Pyle & John L. Earle. 2014. *Neoniphon pencei*, a new species of holocentrid from Rarotonga, Cook Islands. Biodiversity Data Journal.; higherClassification: Animalia; Deuterostomia; Chordata; Craniata; Gnathostomata; Actinopterygii; Beryciformes; Holocentroidei; Holocentridae; Neoniphon; kingdom: Animalia; phylum: Chordata; class: Actinopterygii; order: Beryciformes; family: Holocentridae; genus: Neoniphon; specificEpithet: pencei; taxonRank: species; scientificNameAuthorship: Copus, Pyle & Earle; vernacularName: Pence's Squirrelfish; nomenclaturalCode: ICZN; **Location:** waterBody: Pacific Ocean; islandGroup: Cook Islands; island: Rarotonga; country: Cook Islands; countryCode: CK; locality: E side; Matavera; off Charles J. Boyle's house; verbatimLocality: Cook Islands; Rarotonga; E side; Matavera; off Charles J. Boyle's house; verbatimDepth: 115 m; minimumDepthInMeters: 115; maximumDepthInMeters: 115; decimalLatitude: -21.223798; decimalLongitude: -159.728123; geodeticDatum: WGS 84; coordinateUncertaintyInMeters: 300; georeferenceSources: Google Earth; **Identification:** identifiedBy: Richard L. Pyle; dateIdentified: 2012-08-07; **Event:** samplingProtocol: Spear; eventDate: 2012-07-02; year: 2012; month: 7; day: 2; habitat: small cave near base of vertical drop-off; **Record Level:** modified: 2014-10-09T23:30:00Z; language: en; collectionID: urn:lsid:biocol.org:col:1001; institutionCode: BPBM; collectionCode: Fish; basisOfRecord: PreservedSpecimen
- **Type status:** Paratype. **Occurrence:** catalogNumber: 41196; recordedBy: David F. Pence; individualCount: 1; lifeStage: adult; preparations: 55% Isopropyl; disposition: in collection; associatedSequences: GenBank KJ201926; **Taxon:** taxonID: 43f5caba-6e4b-42bb-8569-8f93d3502de9; scientificNameID: 43f5caba-6e4b-42bb-8569-8f93d3502de9; acceptedNameUsageID: 43f5caba-6e4b-42bb-8569-8f93d3502de9; parentNameUsageID: b047f156-f8da-4ec6-9f64-87345b68a759; originalNameUsageID: 43f5caba-6e4b-42bb-8569-8f93d3502de9; nameAccordingToID: bbdce765-389b-4338-9c36-68def122f4fc; namePublishedInID: bbdce765-389b-4338-9c36-68def122f4fc; scientificName: Neoniphon pencei; acceptedNameUsage: *Neoniphon pencei* Copus, Pyle and Earle, 2014; parentNameUsage: Neoniphon Castelnau, 1875; originalNameUsage: *Neoniphon pencei* Copus, Pyle and Earle, 2014; nameAccordingTo: Copus, Joshua M., Richard L. Pyle & John L. Earle. 2014. *Neoniphon pencei*, a new species of holocentrid from Rarotonga, Cook Islands. Biodiversity Data Journal.; namePublishedIn: Copus, Joshua M., Richard L. Pyle & John L. Earle. 2014. *Neoniphon pencei*, a new species of holocentrid from Rarotonga, Cook Islands. Biodiversity Data Journal.; higherClassification: Animalia; Deuterostomia; Chordata; Craniata; Gnathostomata; Actinopterygii; Beryciformes; Holocentroidei; Holocentridae; Neoniphon; kingdom: Animalia; phylum: Chordata; class: Actinopterygii; order: Beryciformes; family: Holocentridae; genus: Neoniphon; specificEpithet: pencei; taxonRank: species; scientificNameAuthorship: Copus, Pyle & Earle; vernacularName: Pence's Squirrelfish; nomenclaturalCode: ICZN; **Location:** waterBody: Pacific Ocean; islandGroup: Cook Islands; island: Rarotonga; country: Cook Islands; countryCode: CK; locality: E side; Matavera; off Charles J. Boyle's house; verbatimLocality: Cook Islands; Rarotonga; E side; Matavera; off Charles J. Boyle's house; verbatimDepth: 115 m; minimumDepthInMeters: 115; maximumDepthInMeters: 115; decimalLatitude: -21.223798; decimalLongitude: -159.728123; geodeticDatum: WGS 84; coordinateUncertaintyInMeters: 300; georeferenceSources: Google Earth; **Identification:** identifiedBy: Richard L. Pyle; dateIdentified: 2012-08-07; **Event:** samplingProtocol: Spear; eventDate: 2012-07-03; year: 2012; month: 7; day: 3; habitat: small cave near base of vertical drop-off; **Record Level:** modified: 2014-10-09T23:30:00Z; language: en; collectionID: urn:lsid:biocol.org:col:1001; institutionCode: BPBM; collectionCode: Fish; basisOfRecord: PreservedSpecimen
- **Type status:** Paratype. **Occurrence:** catalogNumber: 41196; recordedBy: David F. Pence; individualCount: 1; lifeStage: adult; preparations: 55% Isopropyl; disposition: in collection; associatedSequences: GenBank KJ201926; **Taxon:** taxonID: 43f5caba-6e4b-42bb-8569-8f93d3502de9; scientificNameID: 43f5caba-6e4b-42bb-8569-8f93d3502de9; acceptedNameUsageID: 43f5caba-6e4b-42bb-8569-8f93d3502de9; parentNameUsageID: b047f156-f8da-4ec6-9f64-87345b68a759; originalNameUsageID: 43f5caba-6e4b-42bb-8569-8f93d3502de9; nameAccordingToID: bbdce765-389b-4338-9c36-68def122f4fc; namePublishedInID: bbdce765-389b-4338-9c36-68def122f4fc; scientificName: Neoniphon pencei; acceptedNameUsage: *Neoniphon pencei* Copus, Pyle and Earle, 2014; parentNameUsage: Neoniphon Castelnau, 1875; originalNameUsage: *Neoniphon pencei* Copus, Pyle and Earle, 2014; nameAccordingTo: Copus, Joshua M., Richard L. Pyle & John L. Earle. 2014. *Neoniphon pencei*, a new species of holocentrid from Rarotonga, Cook Islands. Biodiversity Data Journal.; namePublishedIn: Copus, Joshua M., Richard L. Pyle & John L. Earle. 2014. *Neoniphon pencei*, a new species of holocentrid from Rarotonga, Cook Islands. Biodiversity Data Journal.; higherClassification: Animalia; Deuterostomia; Chordata; Craniata; Gnathostomata; Actinopterygii; Beryciformes; Holocentroidei; Holocentridae; Neoniphon; kingdom: Animalia; phylum: Chordata; class: Actinopterygii; order: Beryciformes; family: Holocentridae; genus: Neoniphon; specificEpithet: pencei; taxonRank: species; scientificNameAuthorship: Copus, Pyle & Earle; vernacularName: Pence's Squirrelfish; nomenclaturalCode: ICZN; **Location:** waterBody: Pacific Ocean; islandGroup: Cook Islands; island: Rarotonga; country: Cook Islands; countryCode: CK; locality: E side; Matavera; off Charles J. Boyle's house; verbatimLocality: Cook Islands; Rarotonga; E side; Matavera; off Charles J. Boyle's house; verbatimDepth: 115 m; minimumDepthInMeters: 115; maximumDepthInMeters: 115; decimalLatitude: -21.223798; decimalLongitude: -159.728123; geodeticDatum: WGS 84; coordinateUncertaintyInMeters: 300; georeferenceSources: Google Earth; **Identification:** identifiedBy: Richard L. Pyle; dateIdentified: 2012-08-07; **Event:** samplingProtocol: Spear; eventDate: 2012-07-03; year: 2012; month: 7; day: 3; habitat: small cave near base of vertical drop-off; **Record Level:** modified: 2014-10-09T23:30:00Z; language: en; collectionID: urn:lsid:biocol.org:col:1001; institutionCode: BPBM; collectionCode: Fish; basisOfRecord: PreservedSpecimen
- **Type status:** Paratype. **Occurrence:** catalogNumber: 41196; recordedBy: David F. Pence; individualCount: 1; lifeStage: adult; preparations: 55% Isopropyl; disposition: in collection; associatedSequences: GenBank KJ201926; **Taxon:** taxonID: 43f5caba-6e4b-42bb-8569-8f93d3502de9; scientificNameID: 43f5caba-6e4b-42bb-8569-8f93d3502de9; acceptedNameUsageID: 43f5caba-6e4b-42bb-8569-8f93d3502de9; parentNameUsageID: b047f156-f8da-4ec6-9f64-87345b68a759; originalNameUsageID: 43f5caba-6e4b-42bb-8569-8f93d3502de9; nameAccordingToID: bbdce765-389b-4338-9c36-68def122f4fc; namePublishedInID: bbdce765-389b-4338-9c36-68def122f4fc; scientificName: Neoniphon pencei; acceptedNameUsage: *Neoniphon pencei* Copus, Pyle and Earle, 2014; parentNameUsage: Neoniphon Castelnau, 1875; originalNameUsage: *Neoniphon pencei* Copus, Pyle and Earle, 2014; nameAccordingTo: Copus, Joshua M., Richard L. Pyle & John L. Earle. 2014. *Neoniphon pencei*, a new species of holocentrid from Rarotonga, Cook Islands. Biodiversity Data Journal.; namePublishedIn: Copus, Joshua M., Richard L. Pyle & John L. Earle. 2014. *Neoniphon pencei*, a new species of holocentrid from Rarotonga, Cook Islands. Biodiversity Data Journal.; higherClassification: Animalia; Deuterostomia; Chordata; Craniata; Gnathostomata; Actinopterygii; Beryciformes; Holocentroidei; Holocentridae; Neoniphon; kingdom: Animalia; phylum: Chordata; class: Actinopterygii; order: Beryciformes; family: Holocentridae; genus: Neoniphon; specificEpithet: pencei; taxonRank: species; scientificNameAuthorship: Copus, Pyle & Earle; vernacularName: Pence's Squirrelfish; nomenclaturalCode: ICZN; **Location:** waterBody: Pacific Ocean; islandGroup: Cook Islands; island: Rarotonga; country: Cook Islands; countryCode: CK; locality: E side; Matavera; off Charles J. Boyle's house; verbatimLocality: Cook Islands; Rarotonga; E side; Matavera; off Charles J. Boyle's house; verbatimDepth: 115 m; minimumDepthInMeters: 115; maximumDepthInMeters: 115; decimalLatitude: -21.223798; decimalLongitude: -159.728123; geodeticDatum: WGS 84; coordinateUncertaintyInMeters: 300; georeferenceSources: Google Earth; **Identification:** identifiedBy: Richard L. Pyle; dateIdentified: 2012-08-07; **Event:** samplingProtocol: Spear; eventDate: 2012-07-03; year: 2012; month: 7; day: 3; habitat: small cave near base of vertical drop-off; **Record Level:** modified: 2014-10-09T23:30:00Z; language: en; collectionID: urn:lsid:biocol.org:col:1001; institutionCode: BPBM; collectionCode: Fish; basisOfRecord: PreservedSpecimen
- **Type status:** Paratype. **Occurrence:** catalogNumber: 41196; recordedBy: David F. Pence; individualCount: 1; lifeStage: adult; preparations: 55% Isopropyl; disposition: in collection; associatedSequences: GenBank KJ201926; **Taxon:** taxonID: 43f5caba-6e4b-42bb-8569-8f93d3502de9; scientificNameID: 43f5caba-6e4b-42bb-8569-8f93d3502de9; acceptedNameUsageID: 43f5caba-6e4b-42bb-8569-8f93d3502de9; parentNameUsageID: b047f156-f8da-4ec6-9f64-87345b68a759; originalNameUsageID: 43f5caba-6e4b-42bb-8569-8f93d3502de9; nameAccordingToID: bbdce765-389b-4338-9c36-68def122f4fc; namePublishedInID: bbdce765-389b-4338-9c36-68def122f4fc; scientificName: Neoniphon pencei; acceptedNameUsage: *Neoniphon pencei* Copus, Pyle and Earle, 2014; parentNameUsage: Neoniphon Castelnau, 1875; originalNameUsage: *Neoniphon pencei* Copus, Pyle and Earle, 2014; nameAccordingTo: Copus, Joshua M., Richard L. Pyle & John L. Earle. 2014. *Neoniphon pencei*, a new species of holocentrid from Rarotonga, Cook Islands. Biodiversity Data Journal.; namePublishedIn: Copus, Joshua M., Richard L. Pyle & John L. Earle. 2014. *Neoniphon pencei*, a new species of holocentrid from Rarotonga, Cook Islands. Biodiversity Data Journal.; higherClassification: Animalia; Deuterostomia; Chordata; Craniata; Gnathostomata; Actinopterygii; Beryciformes; Holocentroidei; Holocentridae; Neoniphon; kingdom: Animalia; phylum: Chordata; class: Actinopterygii; order: Beryciformes; family: Holocentridae; genus: Neoniphon; specificEpithet: pencei; taxonRank: species; scientificNameAuthorship: Copus, Pyle & Earle; vernacularName: Pence's Squirrelfish; nomenclaturalCode: ICZN; **Location:** waterBody: Pacific Ocean; islandGroup: Cook Islands; island: Rarotonga; country: Cook Islands; countryCode: CK; locality: E side; Matavera; off Charles J. Boyle's house; verbatimLocality: Cook Islands; Rarotonga; E side; Matavera; off Charles J. Boyle's house; verbatimDepth: 115 m; minimumDepthInMeters: 115; maximumDepthInMeters: 115; decimalLatitude: -21.223798; decimalLongitude: -159.728123; geodeticDatum: WGS 84; coordinateUncertaintyInMeters: 300; georeferenceSources: Google Earth; **Identification:** identifiedBy: Richard L. Pyle; dateIdentified: 2012-08-07; **Event:** samplingProtocol: Spear; eventDate: 2012-07-03; year: 2012; month: 7; day: 3; habitat: small cave near base of vertical drop-off; **Record Level:** modified: 2014-10-09T23:30:00Z; language: en; collectionID: urn:lsid:biocol.org:col:1001; institutionCode: BPBM; collectionCode: Fish; basisOfRecord: PreservedSpecimen
- **Type status:** Paratype. **Occurrence:** catalogNumber: 41196; recordedBy: David F. Pence; individualCount: 1; lifeStage: adult; preparations: 55% Isopropyl; disposition: in collection; associatedSequences: GenBank KJ201926; **Taxon:** taxonID: 43f5caba-6e4b-42bb-8569-8f93d3502de9; scientificNameID: 43f5caba-6e4b-42bb-8569-8f93d3502de9; acceptedNameUsageID: 43f5caba-6e4b-42bb-8569-8f93d3502de9; parentNameUsageID: b047f156-f8da-4ec6-9f64-87345b68a759; originalNameUsageID: 43f5caba-6e4b-42bb-8569-8f93d3502de9; nameAccordingToID: bbdce765-389b-4338-9c36-68def122f4fc; namePublishedInID: bbdce765-389b-4338-9c36-68def122f4fc; scientificName: Neoniphon pencei; acceptedNameUsage: *Neoniphon pencei* Copus, Pyle and Earle, 2014; parentNameUsage: Neoniphon Castelnau, 1875; originalNameUsage: *Neoniphon pencei* Copus, Pyle and Earle, 2014; nameAccordingTo: Copus, Joshua M., Richard L. Pyle & John L. Earle. 2014. *Neoniphon pencei*, a new species of holocentrid from Rarotonga, Cook Islands. Biodiversity Data Journal.; namePublishedIn: Copus, Joshua M., Richard L. Pyle & John L. Earle. 2014. *Neoniphon pencei*, a new species of holocentrid from Rarotonga, Cook Islands. Biodiversity Data Journal.; higherClassification: Animalia; Deuterostomia; Chordata; Craniata; Gnathostomata; Actinopterygii; Beryciformes; Holocentroidei; Holocentridae; Neoniphon; kingdom: Animalia; phylum: Chordata; class: Actinopterygii; order: Beryciformes; family: Holocentridae; genus: Neoniphon; specificEpithet: pencei; taxonRank: species; scientificNameAuthorship: Copus, Pyle & Earle; vernacularName: Pence's Squirrelfish; nomenclaturalCode: ICZN; **Location:** waterBody: Pacific Ocean; islandGroup: Cook Islands; island: Rarotonga; country: Cook Islands; countryCode: CK; locality: E side; Matavera; off Charles J. Boyle's house; verbatimLocality: Cook Islands; Rarotonga; E side; Matavera; off Charles J. Boyle's house; verbatimDepth: 115 m; minimumDepthInMeters: 115; maximumDepthInMeters: 115; decimalLatitude: -21.223798; decimalLongitude: -159.728123; geodeticDatum: WGS 84; coordinateUncertaintyInMeters: 300; georeferenceSources: Google Earth; **Identification:** identifiedBy: Richard L. Pyle; dateIdentified: 2012-08-07; **Event:** samplingProtocol: Spear; eventDate: 2012-07-03; year: 2012; month: 7; day: 3; habitat: small cave near base of vertical drop-off; **Record Level:** modified: 2014-10-09T23:30:00Z; language: en; collectionID: urn:lsid:biocol.org:col:1001; institutionCode: BPBM; collectionCode: Fish; basisOfRecord: PreservedSpecimen
- **Type status:** Paratype. **Occurrence:** catalogNumber: 41196; recordedBy: David F. Pence; individualCount: 1; lifeStage: adult; preparations: 55% Isopropyl; disposition: in collection; associatedSequences: GenBank KJ201926; **Taxon:** taxonID: 43f5caba-6e4b-42bb-8569-8f93d3502de9; scientificNameID: 43f5caba-6e4b-42bb-8569-8f93d3502de9; acceptedNameUsageID: 43f5caba-6e4b-42bb-8569-8f93d3502de9; parentNameUsageID: b047f156-f8da-4ec6-9f64-87345b68a759; originalNameUsageID: 43f5caba-6e4b-42bb-8569-8f93d3502de9; nameAccordingToID: bbdce765-389b-4338-9c36-68def122f4fc; namePublishedInID: bbdce765-389b-4338-9c36-68def122f4fc; scientificName: Neoniphon pencei; acceptedNameUsage: *Neoniphon pencei* Copus, Pyle and Earle, 2014; parentNameUsage: Neoniphon Castelnau, 1875; originalNameUsage: *Neoniphon pencei* Copus, Pyle and Earle, 2014; nameAccordingTo: Copus, Joshua M., Richard L. Pyle & John L. Earle. 2014. *Neoniphon pencei*, a new species of holocentrid from Rarotonga, Cook Islands. Biodiversity Data Journal.; namePublishedIn: Copus, Joshua M., Richard L. Pyle & John L. Earle. 2014. *Neoniphon pencei*, a new species of holocentrid from Rarotonga, Cook Islands. Biodiversity Data Journal.; higherClassification: Animalia; Deuterostomia; Chordata; Craniata; Gnathostomata; Actinopterygii; Beryciformes; Holocentroidei; Holocentridae; Neoniphon; kingdom: Animalia; phylum: Chordata; class: Actinopterygii; order: Beryciformes; family: Holocentridae; genus: Neoniphon; specificEpithet: pencei; taxonRank: species; scientificNameAuthorship: Copus, Pyle & Earle; vernacularName: Pence's Squirrelfish; nomenclaturalCode: ICZN; **Location:** waterBody: Pacific Ocean; islandGroup: Cook Islands; island: Rarotonga; country: Cook Islands; countryCode: CK; locality: E side; Matavera; off Charles J. Boyle's house; verbatimLocality: Cook Islands; Rarotonga; E side; Matavera; off Charles J. Boyle's house; verbatimDepth: 115 m; minimumDepthInMeters: 115; maximumDepthInMeters: 115; decimalLatitude: -21.223798; decimalLongitude: -159.728123; geodeticDatum: WGS 84; coordinateUncertaintyInMeters: 300; georeferenceSources: Google Earth; **Identification:** identifiedBy: Richard L. Pyle; dateIdentified: 2012-08-07; **Event:** samplingProtocol: Spear; eventDate: 2012-07-03; year: 2012; month: 7; day: 3; habitat: small cave near base of vertical drop-off; **Record Level:** modified: 2014-10-09T23:30:00Z; language: en; collectionID: urn:lsid:biocol.org:col:1001; institutionCode: BPBM; collectionCode: Fish; basisOfRecord: PreservedSpecimen
- **Type status:** Paratype. **Occurrence:** catalogNumber: 41196; recordedBy: David F. Pence; individualCount: 1; lifeStage: adult; preparations: 55% Isopropyl; disposition: in collection; associatedSequences: GenBank KJ201926; **Taxon:** taxonID: 43f5caba-6e4b-42bb-8569-8f93d3502de9; scientificNameID: 43f5caba-6e4b-42bb-8569-8f93d3502de9; acceptedNameUsageID: 43f5caba-6e4b-42bb-8569-8f93d3502de9; parentNameUsageID: b047f156-f8da-4ec6-9f64-87345b68a759; originalNameUsageID: 43f5caba-6e4b-42bb-8569-8f93d3502de9; nameAccordingToID: bbdce765-389b-4338-9c36-68def122f4fc; namePublishedInID: bbdce765-389b-4338-9c36-68def122f4fc; scientificName: Neoniphon pencei; acceptedNameUsage: *Neoniphon pencei* Copus, Pyle and Earle, 2014; parentNameUsage: Neoniphon Castelnau, 1875; originalNameUsage: *Neoniphon pencei* Copus, Pyle and Earle, 2014; nameAccordingTo: Copus, Joshua M., Richard L. Pyle & John L. Earle. 2014. *Neoniphon pencei*, a new species of holocentrid from Rarotonga, Cook Islands. Biodiversity Data Journal.; namePublishedIn: Copus, Joshua M., Richard L. Pyle & John L. Earle. 2014. *Neoniphon pencei*, a new species of holocentrid from Rarotonga, Cook Islands. Biodiversity Data Journal.; higherClassification: Animalia; Deuterostomia; Chordata; Craniata; Gnathostomata; Actinopterygii; Beryciformes; Holocentroidei; Holocentridae; Neoniphon; kingdom: Animalia; phylum: Chordata; class: Actinopterygii; order: Beryciformes; family: Holocentridae; genus: Neoniphon; specificEpithet: pencei; taxonRank: species; scientificNameAuthorship: Copus, Pyle & Earle; vernacularName: Pence's Squirrelfish; nomenclaturalCode: ICZN; **Location:** waterBody: Pacific Ocean; islandGroup: Cook Islands; island: Rarotonga; country: Cook Islands; countryCode: CK; locality: E side; Matavera; off Charles J. Boyle's house; verbatimLocality: Cook Islands; Rarotonga; E side; Matavera; off Charles J. Boyle's house; verbatimDepth: 115 m; minimumDepthInMeters: 115; maximumDepthInMeters: 115; decimalLatitude: -21.223798; decimalLongitude: -159.728123; geodeticDatum: WGS 84; coordinateUncertaintyInMeters: 300; georeferenceSources: Google Earth; **Identification:** identifiedBy: Richard L. Pyle; dateIdentified: 2012-08-07; **Event:** samplingProtocol: Spear; eventDate: 2012-07-03; year: 2012; month: 7; day: 3; habitat: small cave near base of vertical drop-off; **Record Level:** modified: 2014-10-09T23:30:00Z; language: en; collectionID: urn:lsid:biocol.org:col:1001; institutionCode: BPBM; collectionCode: Fish; basisOfRecord: PreservedSpecimen
- **Type status:** Paratype. **Occurrence:** catalogNumber: 41196; recordedBy: David F. Pence; individualCount: 1; lifeStage: adult; preparations: 55% Isopropyl; disposition: in collection; associatedSequences: GenBank KJ201926; **Taxon:** taxonID: 43f5caba-6e4b-42bb-8569-8f93d3502de9; scientificNameID: 43f5caba-6e4b-42bb-8569-8f93d3502de9; acceptedNameUsageID: 43f5caba-6e4b-42bb-8569-8f93d3502de9; parentNameUsageID: b047f156-f8da-4ec6-9f64-87345b68a759; originalNameUsageID: 43f5caba-6e4b-42bb-8569-8f93d3502de9; nameAccordingToID: bbdce765-389b-4338-9c36-68def122f4fc; namePublishedInID: bbdce765-389b-4338-9c36-68def122f4fc; scientificName: Neoniphon pencei; acceptedNameUsage: *Neoniphon pencei* Copus, Pyle and Earle, 2014; parentNameUsage: Neoniphon Castelnau, 1875; originalNameUsage: *Neoniphon pencei* Copus, Pyle and Earle, 2014; nameAccordingTo: Copus, Joshua M., Richard L. Pyle & John L. Earle. 2014. *Neoniphon pencei*, a new species of holocentrid from Rarotonga, Cook Islands. Biodiversity Data Journal.; namePublishedIn: Copus, Joshua M., Richard L. Pyle & John L. Earle. 2014. *Neoniphon pencei*, a new species of holocentrid from Rarotonga, Cook Islands. Biodiversity Data Journal.; higherClassification: Animalia; Deuterostomia; Chordata; Craniata; Gnathostomata; Actinopterygii; Beryciformes; Holocentroidei; Holocentridae; Neoniphon; kingdom: Animalia; phylum: Chordata; class: Actinopterygii; order: Beryciformes; family: Holocentridae; genus: Neoniphon; specificEpithet: pencei; taxonRank: species; scientificNameAuthorship: Copus, Pyle & Earle; vernacularName: Pence's Squirrelfish; nomenclaturalCode: ICZN; **Location:** waterBody: Pacific Ocean; islandGroup: Cook Islands; island: Rarotonga; country: Cook Islands; countryCode: CK; locality: E side; Matavera; off Charles J. Boyle's house; verbatimLocality: Cook Islands; Rarotonga; E side; Matavera; off Charles J. Boyle's house; verbatimDepth: 115 m; minimumDepthInMeters: 115; maximumDepthInMeters: 115; decimalLatitude: -21.223798; decimalLongitude: -159.728123; geodeticDatum: WGS 84; coordinateUncertaintyInMeters: 300; georeferenceSources: Google Earth; **Identification:** identifiedBy: Richard L. Pyle; dateIdentified: 2012-08-07; **Event:** samplingProtocol: Spear; eventDate: 2012-07-03; year: 2012; month: 7; day: 3; habitat: small cave near base of vertical drop-off; **Record Level:** modified: 2014-10-09T23:30:00Z; language: en; collectionID: urn:lsid:biocol.org:col:1001; institutionCode: BPBM; collectionCode: Fish; basisOfRecord: PreservedSpecimen
- **Type status:** Paratype. **Occurrence:** catalogNumber: 41196; recordedBy: David F. Pence; individualCount: 1; lifeStage: adult; preparations: 55% Isopropyl; disposition: in collection; associatedSequences: GenBank KJ201926; **Taxon:** taxonID: 43f5caba-6e4b-42bb-8569-8f93d3502de9; scientificNameID: 43f5caba-6e4b-42bb-8569-8f93d3502de9; acceptedNameUsageID: 43f5caba-6e4b-42bb-8569-8f93d3502de9; parentNameUsageID: b047f156-f8da-4ec6-9f64-87345b68a759; originalNameUsageID: 43f5caba-6e4b-42bb-8569-8f93d3502de9; nameAccordingToID: bbdce765-389b-4338-9c36-68def122f4fc; namePublishedInID: bbdce765-389b-4338-9c36-68def122f4fc; scientificName: Neoniphon pencei; acceptedNameUsage: *Neoniphon pencei* Copus, Pyle and Earle, 2014; parentNameUsage: Neoniphon Castelnau, 1875; originalNameUsage: *Neoniphon pencei* Copus, Pyle and Earle, 2014; nameAccordingTo: Copus, Joshua M., Richard L. Pyle & John L. Earle. 2014. *Neoniphon pencei*, a new species of holocentrid from Rarotonga, Cook Islands. Biodiversity Data Journal.; namePublishedIn: Copus, Joshua M., Richard L. Pyle & John L. Earle. 2014. *Neoniphon pencei*, a new species of holocentrid from Rarotonga, Cook Islands. Biodiversity Data Journal.; higherClassification: Animalia; Deuterostomia; Chordata; Craniata; Gnathostomata; Actinopterygii; Beryciformes; Holocentroidei; Holocentridae; Neoniphon; kingdom: Animalia; phylum: Chordata; class: Actinopterygii; order: Beryciformes; family: Holocentridae; genus: Neoniphon; specificEpithet: pencei; taxonRank: species; scientificNameAuthorship: Copus, Pyle & Earle; vernacularName: Pence's Squirrelfish; nomenclaturalCode: ICZN; **Location:** waterBody: Pacific Ocean; islandGroup: Cook Islands; island: Rarotonga; country: Cook Islands; countryCode: CK; locality: E side; Matavera; off Charles J. Boyle's house; verbatimLocality: Cook Islands; Rarotonga; E side; Matavera; off Charles J. Boyle's house; verbatimDepth: 115 m; minimumDepthInMeters: 115; maximumDepthInMeters: 115; decimalLatitude: -21.223798; decimalLongitude: -159.728123; geodeticDatum: WGS 84; coordinateUncertaintyInMeters: 300; georeferenceSources: Google Earth; **Identification:** identifiedBy: Richard L. Pyle; dateIdentified: 2012-08-07; **Event:** samplingProtocol: Spear; eventDate: 2012-07-03; year: 2012; month: 7; day: 3; habitat: small cave near base of vertical drop-off; **Record Level:** modified: 2014-10-09T23:30:00Z; language: en; collectionID: urn:lsid:biocol.org:col:1001; institutionCode: BPBM; collectionCode: Fish; basisOfRecord: PreservedSpecimen
- **Type status:** Paratype. **Occurrence:** catalogNumber: 41196; recordedBy: David F. Pence; individualCount: 1; lifeStage: adult; preparations: 55% Isopropyl; disposition: in collection; associatedSequences: GenBank KJ201926; **Taxon:** taxonID: 43f5caba-6e4b-42bb-8569-8f93d3502de9; scientificNameID: 43f5caba-6e4b-42bb-8569-8f93d3502de9; acceptedNameUsageID: 43f5caba-6e4b-42bb-8569-8f93d3502de9; parentNameUsageID: b047f156-f8da-4ec6-9f64-87345b68a759; originalNameUsageID: 43f5caba-6e4b-42bb-8569-8f93d3502de9; nameAccordingToID: bbdce765-389b-4338-9c36-68def122f4fc; namePublishedInID: bbdce765-389b-4338-9c36-68def122f4fc; scientificName: Neoniphon pencei; acceptedNameUsage: *Neoniphon pencei* Copus, Pyle and Earle, 2014; parentNameUsage: Neoniphon Castelnau, 1875; originalNameUsage: *Neoniphon pencei* Copus, Pyle and Earle, 2014; nameAccordingTo: Copus, Joshua M., Richard L. Pyle & John L. Earle. 2014. *Neoniphon pencei*, a new species of holocentrid from Rarotonga, Cook Islands. Biodiversity Data Journal.; namePublishedIn: Copus, Joshua M., Richard L. Pyle & John L. Earle. 2014. *Neoniphon pencei*, a new species of holocentrid from Rarotonga, Cook Islands. Biodiversity Data Journal.; higherClassification: Animalia; Deuterostomia; Chordata; Craniata; Gnathostomata; Actinopterygii; Beryciformes; Holocentroidei; Holocentridae; Neoniphon; kingdom: Animalia; phylum: Chordata; class: Actinopterygii; order: Beryciformes; family: Holocentridae; genus: Neoniphon; specificEpithet: pencei; taxonRank: species; scientificNameAuthorship: Copus, Pyle & Earle; vernacularName: Pence's Squirrelfish; nomenclaturalCode: ICZN; **Location:** waterBody: Pacific Ocean; islandGroup: Cook Islands; island: Rarotonga; country: Cook Islands; countryCode: CK; locality: E side; Matavera; off Charles J. Boyle's house; verbatimLocality: Cook Islands; Rarotonga; E side; Matavera; off Charles J. Boyle's house; verbatimDepth: 115 m; minimumDepthInMeters: 115; maximumDepthInMeters: 115; decimalLatitude: -21.223798; decimalLongitude: -159.728123; geodeticDatum: WGS 84; coordinateUncertaintyInMeters: 300; georeferenceSources: Google Earth; **Identification:** identifiedBy: Richard L. Pyle; dateIdentified: 2012-08-07; **Event:** samplingProtocol: Spear; eventDate: 2012-07-03; year: 2012; month: 7; day: 3; habitat: small cave near base of vertical drop-off; **Record Level:** modified: 2014-10-09T23:30:00Z; language: en; collectionID: urn:lsid:biocol.org:col:1001; institutionCode: BPBM; collectionCode: Fish; basisOfRecord: PreservedSpecimen
- **Type status:** Paratype. **Occurrence:** catalogNumber: 237596; recordedBy: David F. Pence; individualCount: 1; lifeStage: adult; preparations: 55% Isopropyl; disposition: in collection; associatedSequences: GenBank KJ201926 (C*yt b*); KJ188437 (COI); **Taxon:** taxonID: 43f5caba-6e4b-42bb-8569-8f93d3502de9; scientificNameID: 43f5caba-6e4b-42bb-8569-8f93d3502de9; acceptedNameUsageID: 43f5caba-6e4b-42bb-8569-8f93d3502de9; parentNameUsageID: b047f156-f8da-4ec6-9f64-87345b68a759; originalNameUsageID: 43f5caba-6e4b-42bb-8569-8f93d3502de9; nameAccordingToID: bbdce765-389b-4338-9c36-68def122f4fc; namePublishedInID: bbdce765-389b-4338-9c36-68def122f4fc; scientificName: Neoniphon pencei; acceptedNameUsage: *Neoniphon pencei* Copus, Pyle and Earle, 2014; parentNameUsage: Neoniphon Castelnau, 1875; originalNameUsage: *Neoniphon pencei* Copus, Pyle and Earle, 2014; nameAccordingTo: Copus, Joshua M., Richard L. Pyle & John L. Earle. 2014. *Neoniphon pencei*, a new species of holocentrid from Rarotonga, Cook Islands. Biodiversity Data Journal.; namePublishedIn: Copus, Joshua M., Richard L. Pyle & John L. Earle. 2014. *Neoniphon pencei*, a new species of holocentrid from Rarotonga, Cook Islands. Biodiversity Data Journal.; higherClassification: Animalia; Deuterostomia; Chordata; Craniata; Gnathostomata; Actinopterygii; Beryciformes; Holocentroidei; Holocentridae; Neoniphon; kingdom: Animalia; phylum: Chordata; class: Actinopterygii; order: Beryciformes; family: Holocentridae; genus: Neoniphon; specificEpithet: pencei; taxonRank: species; scientificNameAuthorship: Copus, Pyle & Earle; vernacularName: Pence's Squirrelfish; nomenclaturalCode: ICZN; **Location:** waterBody: Pacific Ocean; islandGroup: Cook Islands; island: Rarotonga; country: Cook Islands; countryCode: CK; locality: E side; Matavera; off Charles J. Boyle's house; verbatimLocality: Cook Islands; Rarotonga; E side; Matavera; off Charles J. Boyle's house; verbatimDepth: 115 m; minimumDepthInMeters: 115; maximumDepthInMeters: 115; decimalLatitude: -21.223798; decimalLongitude: -159.728123; geodeticDatum: WGS 84; coordinateUncertaintyInMeters: 300; georeferenceSources: Google Earth; **Identification:** identifiedBy: Richard L. Pyle; dateIdentified: 2012-08-07; **Event:** samplingProtocol: Spear; eventDate: 2012-07-02; year: 2012; month: 7; day: 2; habitat: small cave near base of vertical drop-off; **Record Level:** modified: 2014-10-09T23:30:00Z; language: en; collectionID: urn:lsid:biocol.org:col:1003; institutionCode: CAS; collectionCode: Fish; basisOfRecord: PreservedSpecimen
- **Type status:** Paratype. **Occurrence:** catalogNumber: 431482; recordedBy: John L. Earle; individualCount: 1; lifeStage: adult; preparations: 55% Isopropyl; disposition: in collection; otherCatalogNumbers: Formerly BPBM 41195; associatedSequences: GenBank KJ201926; **Taxon:** taxonID: 43f5caba-6e4b-42bb-8569-8f93d3502de9; scientificNameID: 43f5caba-6e4b-42bb-8569-8f93d3502de9; acceptedNameUsageID: 43f5caba-6e4b-42bb-8569-8f93d3502de9; parentNameUsageID: b047f156-f8da-4ec6-9f64-87345b68a759; originalNameUsageID: 43f5caba-6e4b-42bb-8569-8f93d3502de9; nameAccordingToID: bbdce765-389b-4338-9c36-68def122f4fc; namePublishedInID: bbdce765-389b-4338-9c36-68def122f4fc; scientificName: Neoniphon pencei; acceptedNameUsage: *Neoniphon pencei* Copus, Pyle and Earle, 2014; parentNameUsage: Neoniphon Castelnau, 1875; originalNameUsage: *Neoniphon pencei* Copus, Pyle and Earle, 2014; nameAccordingTo: Copus, Joshua M., Richard L. Pyle & John L. Earle. 2014. *Neoniphon pencei*, a new species of holocentrid from Rarotonga, Cook Islands. Biodiversity Data Journal.; namePublishedIn: Copus, Joshua M., Richard L. Pyle & John L. Earle. 2014. *Neoniphon pencei*, a new species of holocentrid from Rarotonga, Cook Islands. Biodiversity Data Journal.; higherClassification: Animalia; Deuterostomia; Chordata; Craniata; Gnathostomata; Actinopterygii; Beryciformes; Holocentroidei; Holocentridae; Neoniphon; kingdom: Animalia; phylum: Chordata; class: Actinopterygii; order: Beryciformes; family: Holocentridae; genus: Neoniphon; specificEpithet: pencei; taxonRank: species; scientificNameAuthorship: Copus, Pyle & Earle; vernacularName: Pence's Squirrelfish; nomenclaturalCode: ICZN; **Location:** waterBody: Pacific Ocean; islandGroup: Cook Islands; island: Rarotonga; country: Cook Islands; countryCode: CK; locality: N side; off Avarua Harbor; verbatimLocality: Cook Islands; Rarotonga; N side; off Avarua Harbor; verbatimDepth: 90 m; minimumDepthInMeters: 90; maximumDepthInMeters: 90; decimalLatitude: -21.198947; decimalLongitude: -159.781353; geodeticDatum: WGS 84; coordinateUncertaintyInMeters: 300; georeferenceSources: Google Earth; **Identification:** identifiedBy: Richard L. Pyle; dateIdentified: 2012-08-07; **Event:** samplingProtocol: Quinaldine; eventDate: 2012-06-22; year: 2012; month: 6; day: 22; **Record Level:** modified: 2014-10-09T23:30:00Z; language: en; collectionID: urn:lsid:biocol.org:col:1002; institutionCode: USNM; collectionCode: Fish; basisOfRecord: PreservedSpecimen
- **Type status:** Other material. **Occurrence:** recordedBy: Matt Craig; individualID: NSA128; individualCount: 1; lifeStage: adult; preparations: DMSO; disposition: in collection; associatedSequences: Genbank-KJ188433; **Taxon:** scientificName: Neoniphon sammara; acceptedNameUsage: *Neoniphon sammara* (Forsskål 1775); parentNameUsage: Neoniphon Castelnau, 1875; higherClassification: Animalia; Deuterostomia; Chordata; Craniata; Gnathostomata; Actinopterygii; Beryciformes; Holocentroidei; Holocentridae; Neoniphon; kingdom: Animalia; phylum: Chordata; class: Actinopterygii; order: Beryciformes; family: Holocentridae; genus: Neoniphon; taxonRank: species; vernacularName: Sammara squirelfish; nomenclaturalCode: ICZN; **Location:** waterBody: Indian Ocean; islandGroup: Diego Garcia; country: British Indian Ocean Territory; countryCode: IOT; verbatimLocality: Diego Garcia; **Identification:** identifiedBy: Matt Craig; **Event:** samplingProtocol: Spear; year: 2002-2011; **Record Level:** language: en
- **Type status:** Other material. **Occurrence:** recordedBy: Matt Craig; individualID: NSA129; individualCount: 1; lifeStage: adult; preparations: DMSO; disposition: in collection; associatedSequences: Genbank-KJ188434; **Taxon:** scientificName: Neoniphon sammara; acceptedNameUsage: *Neoniphon sammara* (Forsskål 1775); parentNameUsage: Neoniphon Castelnau, 1875; higherClassification: Animalia; Deuterostomia; Chordata; Craniata; Gnathostomata; Actinopterygii; Beryciformes; Holocentroidei; Holocentridae; Neoniphon; kingdom: Animalia; phylum: Chordata; class: Actinopterygii; order: Beryciformes; family: Holocentridae; genus: Neoniphon; taxonRank: species; vernacularName: Sammara squirelfish; nomenclaturalCode: ICZN; **Location:** waterBody: Indian Ocean; islandGroup: Diego Garcia; country: British Indian Ocean Territory; countryCode: IOT; verbatimLocality: Diego Garcia; **Identification:** identifiedBy: Matt Craig; **Event:** samplingProtocol: Spear; year: 2002-2011; **Record Level:** language: en
- **Type status:** Other material. **Occurrence:** recordedBy: Matt Craig; individualID: NSA130; individualCount: 1; lifeStage: adult; preparations: DMSO; disposition: in collection; associatedSequences: Genbank-KJ188435; **Taxon:** scientificName: Neoniphon sammara; acceptedNameUsage: *Neoniphon sammara* (Forsskål 1775); parentNameUsage: Neoniphon Castelnau, 1875; higherClassification: Animalia; Deuterostomia; Chordata; Craniata; Gnathostomata; Actinopterygii; Beryciformes; Holocentroidei; Holocentridae; Neoniphon; kingdom: Animalia; phylum: Chordata; class: Actinopterygii; order: Beryciformes; family: Holocentridae; genus: Neoniphon; taxonRank: species; vernacularName: Sammara squirelfish; nomenclaturalCode: ICZN; **Location:** waterBody: Indian Ocean; islandGroup: Diego Garcia; country: British Indian Ocean Territory; countryCode: IOT; verbatimLocality: Diego Garcia; **Identification:** identifiedBy: Matt Craig; **Event:** samplingProtocol: Spear; year: 2002-2011; **Record Level:** language: en
- **Type status:** Other material. **Occurrence:** recordedBy: Matt Craig; individualID: NSA131; individualCount: 1; lifeStage: adult; preparations: DMSO; disposition: in collection; associatedSequences: Genbank-KJ188436; **Taxon:** scientificName: Neoniphon sammara; acceptedNameUsage: *Neoniphon sammara* (Forsskål 1775); parentNameUsage: Neoniphon Castelnau, 1875; higherClassification: Animalia; Deuterostomia; Chordata; Craniata; Gnathostomata; Actinopterygii; Beryciformes; Holocentroidei; Holocentridae; Neoniphon; kingdom: Animalia; phylum: Chordata; class: Actinopterygii; order: Beryciformes; family: Holocentridae; genus: Neoniphon; taxonRank: species; vernacularName: Sammara squirelfish; nomenclaturalCode: ICZN; **Location:** waterBody: Indian Ocean; islandGroup: Diego Garcia; country: British Indian Ocean Territory; countryCode: IOT; verbatimLocality: Diego Garcia; **Identification:** identifiedBy: Matt Craig; **Event:** samplingProtocol: Spear; year: 2002-2011; **Record Level:** language: en
- **Type status:** Other material. **Occurrence:** recordedBy: Matt Craig; individualID: NSA132; individualCount: 1; lifeStage: adult; preparations: DMSO; disposition: in collection; associatedSequences: Genbank-KJ188434; **Taxon:** scientificName: Neoniphon sammara; acceptedNameUsage: *Neoniphon sammara* (Forsskål 1775); parentNameUsage: Neoniphon Castelnau, 1875; higherClassification: Animalia; Deuterostomia; Chordata; Craniata; Gnathostomata; Actinopterygii; Beryciformes; Holocentroidei; Holocentridae; Neoniphon; kingdom: Animalia; phylum: Chordata; class: Actinopterygii; order: Beryciformes; family: Holocentridae; genus: Neoniphon; taxonRank: species; vernacularName: Sammara squirelfish; nomenclaturalCode: ICZN; **Location:** waterBody: Indian Ocean; islandGroup: Diego Garcia; country: British Indian Ocean Territory; countryCode: IOT; verbatimLocality: Diego Garcia; **Identification:** identifiedBy: Matt Craig; **Event:** samplingProtocol: Spear; year: 2002-2011; **Record Level:** language: en
- **Type status:** Other material. **Occurrence:** recordedBy: Matt Craig; individualID: NSA133; individualCount: 1; lifeStage: adult; preparations: DMSO; disposition: in collection; associatedSequences: Genbank-KJ188433; **Taxon:** scientificName: Neoniphon sammara; acceptedNameUsage: *Neoniphon sammara* (Forsskål 1775); parentNameUsage: Neoniphon Castelnau, 1875; higherClassification: Animalia; Deuterostomia; Chordata; Craniata; Gnathostomata; Actinopterygii; Beryciformes; Holocentroidei; Holocentridae; Neoniphon; kingdom: Animalia; phylum: Chordata; class: Actinopterygii; order: Beryciformes; family: Holocentridae; genus: Neoniphon; taxonRank: species; vernacularName: Sammara squirelfish; nomenclaturalCode: ICZN; **Location:** waterBody: Indian Ocean; islandGroup: Diego Garcia; country: British Indian Ocean Territory; countryCode: IOT; verbatimLocality: Diego Garcia; **Identification:** identifiedBy: Matt Craig; **Event:** samplingProtocol: Spear; year: 2002-2011; **Record Level:** language: en
- **Type status:** Other material. **Occurrence:** recordedBy: Andrew Gray; individualID: NAU1; individualCount: 1; lifeStage: adult; preparations: DMSO; disposition: in collection; associatedSequences: Genbank-KJ201925; **Taxon:** scientificName: Neoniphon aurolineatus; acceptedNameUsage: *Neoniphon aurolineatus* Liénard 1839; parentNameUsage: Neoniphon Castelnau, 1875; higherClassification: Animalia; Deuterostomia; Chordata; Craniata; Gnathostomata; Actinopterygii; Beryciformes; Holocentroidei; Holocentridae; Neoniphon; kingdom: Animalia; phylum: Chordata; class: Actinopterygii; order: Beryciformes; family: Holocentridae; genus: Neoniphon; taxonRank: species; vernacularName: Yellowstriped squirelfish; nomenclaturalCode: ICZN; **Location:** waterBody: Pacific Ocean; islandGroup: Hawaii; island: Oahu; country: United States; countryCode: USA; verbatimLocality: Hawaii; **Identification:** identifiedBy: Andrew Gray; **Event:** samplingProtocol: Spear; year: 2012; **Record Level:** language: en
- **Type status:** Other material. **Occurrence:** recordedBy: Andrew Gray; individualID: NAU2; individualCount: 1; lifeStage: adult; preparations: DMSO; disposition: in collection; associatedSequences: Genbank-KJ201923; **Taxon:** scientificName: Neoniphon aurolineatus; acceptedNameUsage: *Neoniphon aurolineatus* Liénard 1839; parentNameUsage: Neoniphon Castelnau, 1875; higherClassification: Animalia; Deuterostomia; Chordata; Craniata; Gnathostomata; Actinopterygii; Beryciformes; Holocentroidei; Holocentridae; Neoniphon; kingdom: Animalia; phylum: Chordata; class: Actinopterygii; order: Beryciformes; family: Holocentridae; genus: Neoniphon; taxonRank: species; vernacularName: Yellowstriped squirelfish; nomenclaturalCode: ICZN; **Location:** waterBody: Pacific Ocean; islandGroup: Hawaii; island: Oahu; country: United States; countryCode: USA; verbatimLocality: Hawaii; **Identification:** identifiedBy: Andrew Gray; **Event:** samplingProtocol: Spear; year: 2012; **Record Level:** language: en
- **Type status:** Other material. **Occurrence:** recordedBy: Andrew Gray; individualID: NAU3; individualCount: 1; lifeStage: adult; preparations: DMSO; disposition: in collection; associatedSequences: Genbank-KJ201924; **Taxon:** scientificName: Neoniphon aurolineatus; acceptedNameUsage: *Neoniphon aurolineatus* Liénard 1839; parentNameUsage: Neoniphon Castelnau, 1875; higherClassification: Animalia; Deuterostomia; Chordata; Craniata; Gnathostomata; Actinopterygii; Beryciformes; Holocentroidei; Holocentridae; Neoniphon; kingdom: Animalia; phylum: Chordata; class: Actinopterygii; order: Beryciformes; family: Holocentridae; genus: Neoniphon; taxonRank: species; vernacularName: Yellowstriped squirelfish; nomenclaturalCode: ICZN; **Location:** waterBody: Pacific Ocean; islandGroup: Hawaii; island: Oahu; country: United States; countryCode: USA; verbatimLocality: Hawaii; **Identification:** identifiedBy: Andrew Gray; **Event:** samplingProtocol: Spear; year: 2012; **Record Level:** language: en
- **Type status:** Other material. **Occurrence:** recordedBy: Andrew Gray; individualID: NAU4; individualCount: 1; lifeStage: adult; preparations: DMSO; disposition: in collection; associatedSequences: Genbank-KJ201923; **Taxon:** scientificName: Neoniphon aurolineatus; acceptedNameUsage: *Neoniphon aurolineatus* Liénard 1839; parentNameUsage: Neoniphon Castelnau, 1875; higherClassification: Animalia; Deuterostomia; Chordata; Craniata; Gnathostomata; Actinopterygii; Beryciformes; Holocentroidei; Holocentridae; Neoniphon; kingdom: Animalia; phylum: Chordata; class: Actinopterygii; order: Beryciformes; family: Holocentridae; genus: Neoniphon; taxonRank: species; vernacularName: Yellowstriped squirelfish; nomenclaturalCode: ICZN; **Location:** waterBody: Pacific Ocean; islandGroup: Hawaii; island: Oahu; country: United States; countryCode: USA; verbatimLocality: Hawaii; **Identification:** identifiedBy: Andrew Gray; **Event:** samplingProtocol: Spear; year: 2012; **Record Level:** language: en
- **Type status:** Other material. **Occurrence:** recordedBy: Andrew Gray; individualID: NAU5; individualCount: 1; lifeStage: adult; preparations: DMSO; disposition: in collection; associatedSequences: Genbank-KJ201922; **Taxon:** scientificName: Neoniphon aurolineatus; acceptedNameUsage: *Neoniphon aurolineatus* Liénard 1839; parentNameUsage: Neoniphon Castelnau, 1875; higherClassification: Animalia; Deuterostomia; Chordata; Craniata; Gnathostomata; Actinopterygii; Beryciformes; Holocentroidei; Holocentridae; Neoniphon; kingdom: Animalia; phylum: Chordata; class: Actinopterygii; order: Beryciformes; family: Holocentridae; genus: Neoniphon; taxonRank: species; vernacularName: Yellowstriped squirelfish; nomenclaturalCode: ICZN; **Location:** waterBody: Pacific Ocean; islandGroup: Hawaii; island: Oahu; country: United States; countryCode: USA; verbatimLocality: Hawaii; **Identification:** identifiedBy: Andrew Gray; **Event:** samplingProtocol: Spear; year: 2012; **Record Level:** language: en
- **Type status:** Other material. **Occurrence:** recordedBy: Andrew Gray; individualID: NAU6; individualCount: 1; lifeStage: adult; preparations: DMSO; disposition: in collection; associatedSequences: Genbank-KJ201925; **Taxon:** scientificName: Neoniphon aurolineatus; acceptedNameUsage: *Neoniphon aurolineatus* Liénard 1839; parentNameUsage: Neoniphon Castelnau, 1875; higherClassification: Animalia; Deuterostomia; Chordata; Craniata; Gnathostomata; Actinopterygii; Beryciformes; Holocentroidei; Holocentridae; Neoniphon; kingdom: Animalia; phylum: Chordata; class: Actinopterygii; order: Beryciformes; family: Holocentridae; genus: Neoniphon; taxonRank: species; vernacularName: Yellowstriped squirelfish; nomenclaturalCode: ICZN; **Location:** waterBody: Pacific Ocean; islandGroup: Hawaii; island: Oahu; country: United States; countryCode: USA; verbatimLocality: Hawaii; **Identification:** identifiedBy: Andrew Gray; **Event:** samplingProtocol: Spear; year: 2012; **Record Level:** language: en
- **Type status:** Other material. **Occurrence:** recordedBy: Andrew Gray; individualID: NAU7; individualCount: 1; lifeStage: adult; preparations: DMSO; disposition: in collection; associatedSequences: Genbank-KJ201925; **Taxon:** scientificName: Neoniphon aurolineatus; acceptedNameUsage: *Neoniphon aurolineatus* Liénard 1839; parentNameUsage: Neoniphon Castelnau, 1875; higherClassification: Animalia; Deuterostomia; Chordata; Craniata; Gnathostomata; Actinopterygii; Beryciformes; Holocentroidei; Holocentridae; Neoniphon; kingdom: Animalia; phylum: Chordata; class: Actinopterygii; order: Beryciformes; family: Holocentridae; genus: Neoniphon; taxonRank: species; vernacularName: Yellowstriped squirelfish; nomenclaturalCode: ICZN; **Location:** waterBody: Pacific Ocean; islandGroup: Hawaii; island: Oahu; country: United States; countryCode: USA; verbatimLocality: Hawaii; **Identification:** identifiedBy: Andrew Gray; **Event:** samplingProtocol: Spear; year: 2012; **Record Level:** language: en
- **Type status:** Other material. **Occurrence:** recordedBy: Matt Craig; individualID: NOP1; individualCount: 1; lifeStage: adult; preparations: DMSO; disposition: in collection; associatedSequences: Genbank-KJ188432; **Taxon:** scientificName: Neoniphon opercularis; acceptedNameUsage: *Neoniphon opercularis* Valenciennes 1831; parentNameUsage: Neoniphon Castelnau, 1875; higherClassification: Animalia; Deuterostomia; Chordata; Craniata; Gnathostomata; Actinopterygii; Beryciformes; Holocentroidei; Holocentridae; Neoniphon; kingdom: Animalia; phylum: Chordata; class: Actinopterygii; order: Beryciformes; family: Holocentridae; genus: Neoniphon; taxonRank: species; vernacularName: Blackfin squirelfish; nomenclaturalCode: ICZN; **Location:** waterBody: Pacific Ocean; islandGroup: Society; island: Moorea; country: French Polynesia; countryCode: PYF; verbatimLocality: Society; **Identification:** identifiedBy: Matt Craig; **Event:** samplingProtocol: Spear; year: 2002-2011; **Record Level:** language: en
- **Type status:** Other material. **Occurrence:** recordedBy: Matt Craig; individualID: NOP2; individualCount: 1; lifeStage: adult; preparations: DMSO; disposition: in collection; associatedSequences: Genbank-KJ188432; **Taxon:** scientificName: Neoniphon opercularis; acceptedNameUsage: *Neoniphon opercularis* Valenciennes 1831; parentNameUsage: Neoniphon Castelnau, 1875; higherClassification: Animalia; Deuterostomia; Chordata; Craniata; Gnathostomata; Actinopterygii; Beryciformes; Holocentroidei; Holocentridae; Neoniphon; kingdom: Animalia; phylum: Chordata; class: Actinopterygii; order: Beryciformes; family: Holocentridae; genus: Neoniphon; taxonRank: species; vernacularName: Blackfin squirelfish; nomenclaturalCode: ICZN; **Location:** waterBody: Pacific Ocean; islandGroup: Society; island: Moorea; country: French Polynesia; countryCode: PYF; verbatimLocality: Society; **Identification:** identifiedBy: Matt Craig; **Event:** samplingProtocol: Spear; year: 2002-2011; **Record Level:** language: en
- **Type status:** Other material. **Occurrence:** recordedBy: Matt Craig; individualID: NAR1; individualCount: 1; lifeStage: adult; preparations: DMSO; disposition: in collection; associatedSequences: Genbank-KJ188431; **Taxon:** scientificName: Neoniphon argenteus; acceptedNameUsage: *Neoniphon argenteus* Valenciennes 1831; parentNameUsage: Neoniphon Castelnau, 1875; higherClassification: Animalia; Deuterostomia; Chordata; Craniata; Gnathostomata; Actinopterygii; Beryciformes; Holocentroidei; Holocentridae; Neoniphon; kingdom: Animalia; phylum: Chordata; class: Actinopterygii; order: Beryciformes; family: Holocentridae; genus: Neoniphon; taxonRank: species; vernacularName: Clearfin squirelfish; nomenclaturalCode: ICZN; **Location:** waterBody: Pacific Ocean; islandGroup: Line; island: Kiritimati; country: Republic of Kiritimati; countryCode: KIR; verbatimLocality: Line; **Identification:** identifiedBy: Matt Craig; **Event:** samplingProtocol: Spear; year: 2002-2011; **Record Level:** language: en
- **Type status:** Other material. **Occurrence:** recordedBy: Matt Craig; individualID: NAR2; individualCount: 1; lifeStage: adult; preparations: DMSO; disposition: in collection; associatedSequences: Genbank-KJ188431; **Taxon:** scientificName: Neoniphon argenteus; acceptedNameUsage: *Neoniphon argenteus* Valenciennes 1831; parentNameUsage: Neoniphon Castelnau, 1875; higherClassification: Animalia; Deuterostomia; Chordata; Craniata; Gnathostomata; Actinopterygii; Beryciformes; Holocentroidei; Holocentridae; Neoniphon; kingdom: Animalia; phylum: Chordata; class: Actinopterygii; order: Beryciformes; family: Holocentridae; genus: Neoniphon; taxonRank: species; vernacularName: Clearfin squirelfish; nomenclaturalCode: ICZN; **Location:** waterBody: Pacific Ocean; islandGroup: Line; island: Kiritimati; country: Republic of Kiritimati; countryCode: KIR; verbatimLocality: Line; **Identification:** identifiedBy: Matt Craig; **Event:** samplingProtocol: Spear; year: 2002-2011; **Record Level:** language: en
- **Type status:** Other material. **Occurrence:** recordedBy: Matt Craig; individualID: NAR3; individualCount: 1; lifeStage: adult; preparations: DMSO; disposition: in collection; associatedSequences: Genbank-KJ188431; **Taxon:** scientificName: Neoniphon argenteus; acceptedNameUsage: *Neoniphon argenteus* Valenciennes 1831; parentNameUsage: Neoniphon Castelnau, 1875; higherClassification: Animalia; Deuterostomia; Chordata; Craniata; Gnathostomata; Actinopterygii; Beryciformes; Holocentroidei; Holocentridae; Neoniphon; kingdom: Animalia; phylum: Chordata; class: Actinopterygii; order: Beryciformes; family: Holocentridae; genus: Neoniphon; taxonRank: species; vernacularName: Clearfin squirelfish; nomenclaturalCode: ICZN; **Location:** waterBody: Pacific Ocean; islandGroup: Line; island: Kiritimati; country: Republic of Kiritimati; countryCode: KIR; verbatimLocality: Line; **Identification:** identifiedBy: Matt Craig; **Event:** samplingProtocol: Spear; year: 2002-2011; **Record Level:** language: en
- **Type status:** Other material. **Occurrence:** recordedBy: Matt Craig; individualID: NAR4; individualCount: 1; lifeStage: adult; preparations: DMSO; disposition: in collection; associatedSequences: Genbank-KJ188431; **Taxon:** scientificName: Neoniphon argenteus; acceptedNameUsage: *Neoniphon argenteus* Valenciennes 1831; parentNameUsage: Neoniphon Castelnau, 1875; higherClassification: Animalia; Deuterostomia; Chordata; Craniata; Gnathostomata; Actinopterygii; Beryciformes; Holocentroidei; Holocentridae; Neoniphon; kingdom: Animalia; phylum: Chordata; class: Actinopterygii; order: Beryciformes; family: Holocentridae; genus: Neoniphon; taxonRank: species; vernacularName: Clearfin squirelfish; nomenclaturalCode: ICZN; **Location:** waterBody: Pacific Ocean; islandGroup: Line; island: Kiritimati; country: Republic of Kiritimati; countryCode: KIR; verbatimLocality: Line; **Identification:** identifiedBy: Matt Craig; **Event:** samplingProtocol: Spear; year: 2002-2011; **Record Level:** language: en
- **Type status:** Other material. **Occurrence:** recordedBy: Matt Craig; individualID: NAR5; individualCount: 1; lifeStage: adult; preparations: DMSO; disposition: in collection; associatedSequences: Genbank-KJ188431; **Taxon:** scientificName: Neoniphon argenteus; acceptedNameUsage: *Neoniphon argenteus* Valenciennes 1831; parentNameUsage: Neoniphon Castelnau, 1875; higherClassification: Animalia; Deuterostomia; Chordata; Craniata; Gnathostomata; Actinopterygii; Beryciformes; Holocentroidei; Holocentridae; Neoniphon; kingdom: Animalia; phylum: Chordata; class: Actinopterygii; order: Beryciformes; family: Holocentridae; genus: Neoniphon; taxonRank: species; vernacularName: Clearfin squirelfish; nomenclaturalCode: ICZN; **Location:** waterBody: Pacific Ocean; islandGroup: Line; island: Kiritimati; country: Republic of Kiritimati; countryCode: KIR; verbatimLocality: Line; **Identification:** identifiedBy: Matt Craig; **Event:** samplingProtocol: Spear; year: 2002-2011; **Record Level:** language: en
- **Type status:** Other material. **Occurrence:** recordedBy: Matt Craig; individualID: NAR6; individualCount: 1; lifeStage: adult; preparations: DMSO; disposition: in collection; associatedSequences: Genbank-KJ188431; **Taxon:** scientificName: Neoniphon argenteus; acceptedNameUsage: *Neoniphon argenteus* Valenciennes 1831; parentNameUsage: Neoniphon Castelnau, 1875; higherClassification: Animalia; Deuterostomia; Chordata; Craniata; Gnathostomata; Actinopterygii; Beryciformes; Holocentroidei; Holocentridae; Neoniphon; kingdom: Animalia; phylum: Chordata; class: Actinopterygii; order: Beryciformes; family: Holocentridae; genus: Neoniphon; taxonRank: species; vernacularName: Clearfin squirelfish; nomenclaturalCode: ICZN; **Location:** waterBody: Pacific Ocean; islandGroup: Line; island: Kiritimati; country: Republic of Kiritimati; countryCode: KIR; verbatimLocality: Line; **Identification:** identifiedBy: Matt Craig; **Event:** samplingProtocol: Spear; year: 2002-2011; **Record Level:** language: en
- **Type status:** Other material. **Occurrence:** recordedBy: Matt Craig; individualID: NMA1; individualCount: 1; lifeStage: adult; preparations: DMSO; disposition: in collection; associatedSequences: Genbank-KJ201921; **Taxon:** scientificName: Neoniphon marianus; acceptedNameUsage: *Neoniphon marianus* Cuvier 1829; parentNameUsage: Neoniphon Castelnau, 1875; higherClassification: Animalia; Deuterostomia; Chordata; Craniata; Gnathostomata; Actinopterygii; Beryciformes; Holocentroidei; Holocentridae; Neoniphon; kingdom: Animalia; phylum: Chordata; class: Actinopterygii; order: Beryciformes; family: Holocentridae; genus: Neoniphon; taxonRank: species; vernacularName: Longjaw squirelfish; nomenclaturalCode: ICZN; **Location:** waterBody: Atlantic Ocean; islandGroup: Bahamas; island: Bahamas; country: Commonwelth of the Bahamas; countryCode: BHS; verbatimLocality: Bahamas; **Identification:** identifiedBy: Casey Benkwitt; **Event:** samplingProtocol: Spear; year: 2013; **Record Level:** language: en

#### Description

Dorsal rays XI, 13, the last ray branched to base; anal rays IV,9, the last ray branched to base; principal caudal rays 17, the upper and lower unbranched; upper procurrent caudal rays 7, the first spinous, the last slender and segmented; lower procurrent caudal rays 6, the first 5 spinous, the last slender and segmented; pectoral rays 14, the uppermost rudimentary, the second and lowermost unbranched; pelvic rays I,7; lateral line scales 49 (48-52); scales above lateral line to base of dorsal spines 5; scales below lateral line to origin of anal fin 6 (6-7); oblique rows of scales on cheek 5; vertical row of 9 (8-10) scales on opercle; gill rakers 6+13 (6-7+13); vertebrae 25; body depth 3.2 (2.9-3.3) in SL; head length 2.8 (2.6-2.9) in SL; snout length 3.5 (3.4-3.8) in head length; orbit diameter 3.0 (2.6-3.0) in head length; interorbital width 4.8 (4.1-4.8) in body depth; upper-jaw length 2.3 (2.3-2.6) in head length; preopercular spine 2.2 (2.0-2.9) in orbit diameter; caudal peduncle depth 3.9 (3.7-4.3) in body depth; caudal peduncle length 7.3 (6.5-7.7) in SL, predorsal length 2.7 (2.4-2.7) in SL; preanal length 1.3 (1.2-1.3) in SL; prepelvic length 2.4 (2.4-2.6) in SL; first dorsal spine 3.5 (3.3-4.1) in head length; third dorsal spine longest, 2.3 (2.0-2.8) in head length; first anal spine 29.5 (19.0-29.5) in head length; second anal spine 9.0 (7.2-9.0) in head length; third anal spine 1.3 (1.1-1.3) in head length; fourth anal spine 2.0 (1.7-2.0) in head length; longest anal ray 2.0 (1.9-2.2) in head length; caudal-fin length 5.0 (3.3-5.2) in SL; caudal concavity 2.9 (2.0-2.9) in head length; pectoral-fin length 4.1 (3.6-4.2) in SL; pelvic-spine length 2.3 (2.2-2.5) in head length; pelvic-fin length 4.9 (4.3-4.9) in SL.

**Figure 1.**
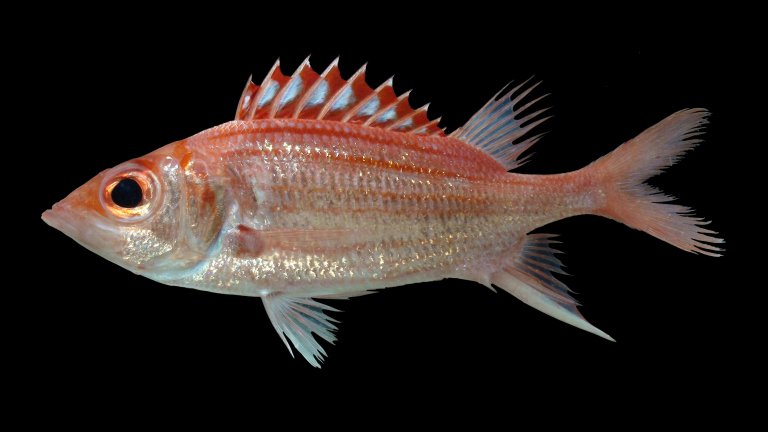
Holotype of *Neoniphon pencei*, BPBM 41197, Rarotonga, Cook Islands. Photo: Richard Pyle and Brian Greene.

**Figure 2.**
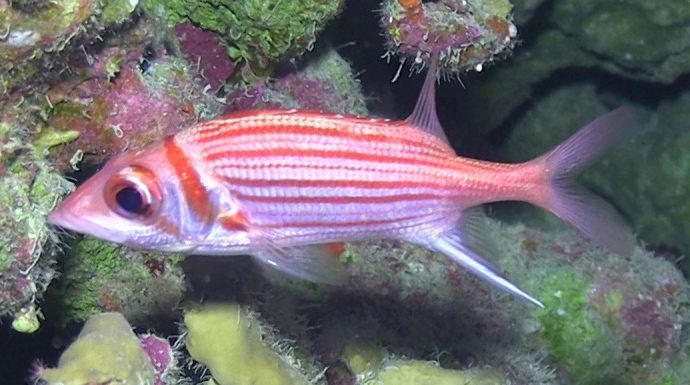
*Neoniphon pencei* at approximately 70 m in Rarotonga, Cook Islands. Cropped from a video frame taken by J.L. Earle.

**Figure 3.**
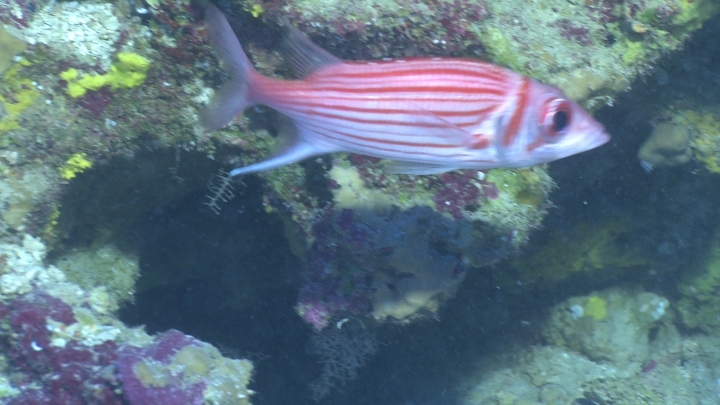
An apparent *Neoniphon pencei* at approximately 90 m in Moorea. Cropped from a video frame taken by R.K. Whitton.

Color in life (Figs 1, 2, 3): Body silvery white with an orange-red tint above lateral line. Scales above lateral line with orange-red borders. Approximately eleven red to orange-red stripes following and sometimes bisecting scales of each horizontal scale row, width of stripes on body alternating between very narrow stripes and stripes over three times wider, except for two consecutive wide stripes, numbers six and seven counted ventrally from the thin dorsal-most stripe, the eleventh ventral-most stripe thin and barely visible on some specimens. Preopercle silvery white with a narrow orange-red posterior border, faint on some specimens. Opercle, nape and interorbital space orange-red. Prominent red bar of less than pupil width extending across nape to level of pectoral axil when viewed underwater. Pectoral axil orange-red. Dorsal fin spines and rays light orange red. Membranes of spinous portion of dorsal fin red with white tips and a white semicircular spot encompassing the middle vertical third of each membrane, its greatest length along the preceding anterior spine and not extending to the posterior spine. Some specimens without a white spot on the first membrane. Soft dorsal fin, pectoral fin, anal fin and pelvic fins with transparent membranes, except anal fin with translucent white membrane between longest spines. Pectoral fin and pelvic fin rays with faint pinkish tint. Anal fin spines white with a faint orange tint on some specimens. Anal fin rays orange-red. Caudal fin rays orange-red, faint on inner rays, membranes translucent white.

Color in alcohol: Body pale yellowish-white. Narrow orange-tan stripes bisecting scales of horizontal scale rows, except for lateral line scale row, the stripes above lateral line faint, barely visible on some specimens. Preopercle white with narrow yellow-tan border. Opercle, nape and interorbital space yellow-tan. Spinous dorsal fin membranes translucent with a white tint. Soft dorsal fin, anal fin pectoral fin and pelvic fin membranes transparent. Caudal fin with orange-tan blotch on upper and lower base, extending faintly on to upper and lower rays. Middle third of caudal fin rays and membranes transparent.

#### Diagnosis

Dorsal rays XI,13; anal rays IV,9; pectoral rays 14; lateral-line scales 48-52 (usually 49); scales above lateral line to base of dorsal spines 5; scales below lateral line to base of anal fin 6-7; oblique rows of scales on cheek 5; gill rakers 6-7+13 (usually 6+13); body slender, the depth 2.9-3.3 in SL; head length 2.6-2.9 in SL; orbit diameter 2.7-3.0 in head length; interorbital width 4.1-4.8 in body depth; upper jaw length 2.3-2.6 in head; lower jaw strongly protruding; preopercular spine 2.0-2.9 of orbit diameter; first dorsal spine 3.3-4.1 in head length; last dorsal spine shortest; third anal spine the longest, its length 1.1-1.3 in head length; body red with white stripes dorsally, front edge of pelvic and anal fins white, white on base of dorsal fin; reaches 24cm.

#### Etymology

Named for David F. Pence, Dive Safety Officer for the University of Hawai'i, a member of the deep diving team that discovered this species, in recognition of his efforts to collect the type specimens.

#### Distribution

All type specimens of *N. pencei* were collected at Rarotonga, Cook Islands. An individual *Neoniphon* closely matching the life colors of *N. pencei* (and different from all other known species) was captured on video by Robert K. Whitton at a depth of 90 m at Moorea, in February 2012 (Fig. 3). It is likely that the species is more broadly distributed throughout the southeastern tropical Pacific, but has escaped noticed due to insufficient collecting activities at mesophotic depths in this region.

## Analysis

**Genetic results**

**Figure 4.**
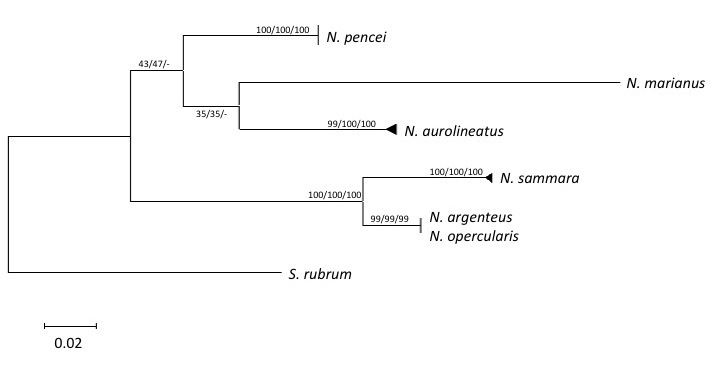
Maximum likelihood phylogenetic reconstruction for the genus *Neoniphon* based on *Cyt b* sequences from 35 individuals, yielding 12 unique haplotypes, rooted with *Sargocentron rubrum*. Branch support values are Maximum Likelihood, Neighbor-Joining, and Maximum Parsimony bootstrap percent values respectively. Triangles at branch termini represent multiple haplotypes; vertical bars at branch termini represent multiple individuals with identical haplotypes.

After alignment and editing, a 377-bp partial sequence of *Cyt b* was obtained for all thirty-five *Neoniphon* samples, resulting in twelve unique haplotypes. All three phylogenetic methods used resulted in congruent tree topologies and are presented as a Maximum Likelihood reconstruction (Fig. 4). Phylogenetic reconstruction recovered strong support for clades corresponding to known *Neoniphon* species. The species *N. pencei* showed strong clade support (100% bootstrap support for all three methods) for belonging to a single clade distinct from currently described *Neoniphon* species. There was not enough signal to resolve the sister relationship between some members within the genus *Neoniphon*; however, this description is not necessary for the goals of this study. *Neoniphon pencei* shows 9-12.5% uncorrected sequence divergence and 34-47 mutations between all other known *Neoniphon* species and posesses 8 diagnostic sites unique from all other species of *Neoniphon* within this this region of *Cyt b*. This is consistent with species level sequence divergence found in other fish taxa (Bellwood et al. 2004, Fessler and Westneat 2007, Randall and Rocha 2009, Rocha 2004, Rocha et al. 2008).

## Discussion

Most recent authors who have reported on *Neoniphon* (e.g., Randall and Heemstra 1985, Randall and Heemstra 1986, Kotlyar 1996, Kotlyar 1998, Randall and Greenfield 1999, Greenfield 2003, Satapoomin 2009) consider it to be a valid genus (a senior synonym of *Flammeo*), distinct from other genera in the subfamily Holocentrinae (particularly *Sargocentron*; Fowler 1904), primarily on the basis of the position of the last dorsal-fin spine (relative to the penultimate dorsal-fin spine and first dorsal-fin ray), and the protruding lower jaw in species of *Neoniphon* (Randall and Heemstra 1985). A more recent phylogenetic analysis of holocentrids by Dornburg et al. (2012), however, reported evidence that *Sargocentron* and *Neoniphon* are paraphyletic. Specifically, they found that four of the five species of *Neoniphon* (they did not include *N. aurolineatus* in their analyses) cluster among several subclades that include nine of the seventeen species of *Sargocentron* they analyzed (*S. coruscum*, *S. diadema*, *S. inaequalis*, *S. ittodai*, *S. microstoma*, *S. punctatissimum*, *S. suborbitalis* [=suborbitale], *S. vexillarium* and *S. xantherythrum*). The other eight species of *Sargocentron* they analyzed (*S. caudimaculatum*, *S. cornutum*, *S. melanospilos*, *S. praslin*, *S. rubrum*, *S. seychellense*, *S. spiniferum* and *S. tiere*) form a separate clade (their "*Sargocentron* group 1"). They argue that the characters used to differentiate these species are ecologically plastic and therefore current relationships represent ecotypes rather than their evolutionary relationships. We acknowledge the results of this study and welcome a new comprehensive analysis of the entire Holocentrinae in light of new genetic evidence. However, in the absence of observed morphological characters that are consistent with the genetic results, we choose to retain these six species within the genus *Neoniphon*, to the exclusion of *Sargocentron*, thereby maintaining nomenclatural stability. *Neoniphon pencei* clearly differs from all species placed in the genus *Sargocentron* on the basis of a closer association of the last dorsal-fin spine with the first soft-ray rather than the penultimate spine and the strongly protruding lower jaw (Randall and Heemstra 1985) as well as life color.

﻿Meristic data of the type specimens of *Neoniphon pencei* are included in Table 1, and proportional measurements are included in Table 2. *Neoniphon pencei* is distinctive from all other species of holocentrids, both morphologically and genetically. Table 3 summarizes morphological differences between *N. pencei* and other species in the genus. It differs most substantially from all other *Neoniphon* in number of lateral line scales (48-52, compared with 38-47 among all other species), number of scales above the lateral line to the origin of the dorsal fin (5, compared with 2.5-3.5) and number of scales below the lateral line to the origin of the anal fin (6-7, compared with 7-9). It also differs from *N. aurolineatus*, *N. opercularis*, and *N. argenteus* in proportional length of the upper-jaw (2.3-2.6 in head length, compared with 2.0-2.3), and proportional length of of the third and fourth anal spines (1.1-1.3 and 1.7-2.0, compared with 1.4-1.9 and 1.9-2.7, respectively). It is further distinguished from *N. aurolineatus* in total number of gill rakers (19-20, compared with 15-17); from *N. opercularis* in head length (2.6-2.9 in SL, compared with 2.9-3.1), orbit diameter (1.2-1.4 in head length, compared with 3.0-3.5), snout length (1.2-1.4 in orbit diameter, compared with 1.2-1.5), and interorbital width (1.7-1.9 in orbit diameter, compared with 1.2-1.5); from *N. argenteus* in number of pectoral rays (14, compared with 12-13), interorbital width (1.7-1.9 in orbit diameter, compared with 1.2-1.7), and first dorsal-spine length (3.3-4.1 in head length, compared with 2.4-3.1); and from *N. sammara* in number of soft dorsal-fin rays (13, compared with 11-12), interorbital width (1.7-1.9 in orbit diameter, compared with 1.3-1.6), and first dorsal-spine length (3.3-4.1 in head length, compared with 2.2-3.0). In addition to these morphometric characters, *N. pencei* differs from all other species of *Neoniphon* in life color, particularly in the pattern of white spots on the dorsal fin and overall body color, and the lack of yellow coloration on the body (as in *N. aurolineatus* and *N. marianus*). Genetically, it differs in its *Cyt b* sequence from *N. argenteus* by 9.8%, *N. aurolineatus* by 9-9.6%, *N. marianus* by 11.7%, *N. opercularis* by 9.8%, and *N. sammara* by 12-12.5%.

*Neoniphon pencei* appears most similar to *N. aurolineatus* and *N. marianus*, based on having the the fewest number of differences in morphometrics, greatest genetic similarity, and most similar aspects of life coloration with these two species. It is also similar to *N. aurolineatus* in the depth and habitat it occupies. However, the differences between *N. pencei* and these two species as noted above clearly warrant recognition of *N. pencei* as a distinct species. A more comprehensive phylogenetic analysis of the species of *Neoniphon* and related genera based on both morphology and genetics (with verified voucher specimens) is beyond the scope of this work.

## Supplementary Material

XML Treatment for Neoniphon pencei

## Figures and Tables

**Table 1.** Meristic data of the type specimens of *Neoniphon pencei*.

	**Holo-** **type**	**Paratypes**											
	BPBM 41197	BPBM 41196	BPBM 41196	BPBM 41196	BPBM 41196	BPBM 41196	BPBM 41196	BPBM 41196	BPBM 41196	BPBM 41196	BPBM 41196	USNM 431482	CAS 237596
	175	197	172	170	159	157	162	150	160	160	135	165	132
**Dorsal** **Fin** **Rays**	XI,13	XI,13	XI,13	XI,13	XI,13	XI,13	XI,13	XI,13	XI,13	XI,13	XI,13	XI,13	XI,13
**Anal** **Fin** **Rays**	IV,9	IV,9	IV,9	IV,9	IV,9	IV,9	IV,9	IV,9	IV,9	IV,9	IV,9	IV,9	IV,9
**Pectoral** **Fin** **Rays**	14	14	14	14	14	14	14	14	14	14	14	14	14
**Pelvic** **Fin Rays**	I,7	I,7	I,7	I,7	I,7	I,7	I,7	I,7	I,7	I,7	I,7	I,7	I,7
**Principal** **Caudal** **Rays**	9+8	9+8	9+8	9+8	9+8	9+8	9+8	9+8	9+8	dama- ged	9+8	9+8	9+8
**Upper** **Pro-** **current** **Caudal** **Rays**	7	7	7	7	7	7	7	7	7	dama- ged	7	7	7
**Lower** **Pro-** **current** **Caudal** **Rays**	6	6	6	6	6	6	6	6	6	dama- ged	6	6	6
**Lateral** **Line** **Scales**	49	49	49	49	49	49	49	48	49	dama- ged	49	52	49
**Scales** **Above** **Lateral** **Line**	5	5	5	5	5	5	5	5	5	5	5	5	5
**Scales** **Below** **Lateral** **Line**	7	7	6	7	6	6	6	6	6	6	6	6	7
**Cheek** **Scales**	5	5	5	5	5	5	5	5	5	5	5	5	5
**Opercle** **Scales**	9	9	10	10	9	10	8	9	10	10	9	9	9
**Gill** **Rakers**	6+13	7+13	6+13	6+13	6+13	6+13	6+13	6+13	7+13	6+13	7+13	6+13	6+13
**Verte-** **brate**	25	25	25	25	25	25	25	25	25	25	25	25	25

**Table 2.** Proportional measurements of type specimens of *Neoniphon pencei* as percentages of standard length^1^, head length^2^, orbit diameter^3^, or body depth^4^.

	**Holo-** **type**	**Paratypes**											
	BPBM 41197	BPBM 41196	BPBM 41196	BPBM 41196	BPBM 41196	BPBM 41196	BPBM 41196	BPBM 41196	BPBM 41196	BPBM 41196	BPBM 41196	USNM 431482	CAS 237596
**Standard** **length** **(mm)**	175	197	172	170	170	157	162	150	160	160	135	165	132
**Body** **depth^1^**	3.15	3.30	3.04	3.06	3.09	2.90	3.16	3.06	3.02	3.06	3.16	3.08	3.03
**Head** **length^1^**	2.82	2.93	2.77	2.79	2.83	2.60	2.72	2.69	2.74	2.70	2.81	2.84	2.73
**Snout** **length^2^**	3.54	3.60	3.70	3.49	3.69	3.54	3.72	3.78	3.66	3.43	3.76	3.63	3.66
**Orbit** **diameter^2^**	3.02	2.80	2.73	2.94	2.82	3.01	2.90	2.75	2.72	2.96	2.63	2.76	2.73
**Inter-** **orbital** **width^4^**	4.83	4.69	4.11	4.72	4.78	4.70	4.18	4.45	4.51	4.35	4.38	4.46	4.61
**Upper-** **jaw** **length^2^**	2.30	2.32	2.38	2.35	2.35	2.41	2.45	2.59	2.29	2.42	2.34	2.27	2.31
**Preoper-** **cular** **spine^3^**	2.16	2.00	2.39	2.44	2.66	2.86	2.34	2.53	1.95	broken	2.61	2.00	2.54
**Caudal-** **peduncle** **depth^4^**	3.90	3.92	4.04	3.83	3.79	4.00	3.73	3.77	3.93	dama- ged	4.28	4.12	4.17
**Caudal** **peduncle** **length^1^**	7.30	6.74	7.24	7.23	7.16	6.82	6.48	6.45	6.81	dama- ged	7.01	6.88	7.68
**Predorsal** **length^1^**	2.66	2.72	2.52	2.64	2.54	2.43	2.58	2.53	2.43	2.46	2.53	2.61	2.52
**Preanal** **length^1^**	1.32	1.34	1.26	1.34	1.29	1.27	1.31	1.21	1.30	1.25	1.23	1.40	1.31
**Prepelvic** **length^1^**	2.44	2.48	2.51	2.48	2.64	2.43	2.57	2.49	2.60	2.46	2.52	2.62	2.48
**First** **dorsal** **spine^2^**	3.50	4.14	3.99	broken	3.66	4.09	3.65	3.54	3.86	3.65	3.45	3.84	3.33
**Longest** **dorsal** **spine^2^**	3.34	2.44	2.30	2.37	2.31	2.37	2.25	2.03	2.40	2.68	2.24	2.85	2.21
**First** **anal** **spine^2^**	29.5	25.0	25.2	23.2	27.4	23.6	23.2	25.2	26.0	22.3	24.9	25.7	19.1
**Second** **anal** **spine^2^**	9.00	8.17	8.09	7.24	8.52	8.25	8.26	7.84	8.29	7.66	7.88	7.95	7.34
**Third** **anal** **spine^2^**	1.34	1.24	1.21	1.20	1.25	1.22	1.26	1.16	1.13	1.17	1.13	1.18	1.16
**Fourth** **anal** **spine^2^**	1.98	1.97	1.80	1.89	1.89	1.93	2.03	2.00	1.74	1.99	1.71	1.89	1.88
**Longest** **anal** **ray^2^**	2.05	2.05	1.92	1.96	2.0	2.09	2.13	2.12	1.94	2.21	1.89	2.12	1.97
**Caudal-** **fin** **length^1^**	5.00	5.18	4.30	4.25	4.25	3.65	3.77	3.33	3.76	4.00	4.50	4.71	3.53
**Caudal** **concavity^2^**	2.88	2.54	2.23	2.30	2.47	1.98	2.20	2.45	2.21	dama- ged	2.78	2.90	2.16
**Pectoral-** **fin** **length^1^**	4.12	4.08	4.14	4.20	4.0	3.63	3.90	3.92	4.05	3.79	3.88	4.29	4.11
**Pelvic-** **spine** **length^2^**	2.32	2.26	2.21	2.26	2.31	2.51	2.33	2.23	2.29	2.37	2.29	2.47	2.43
**Pelvic-** **fin** **length^1^**	4.93	4.83	4.74	4.42	4.59	4.30	4.63	4.41	4.44	4.54	4.58	4.93	4.57

**Table 3.** Comparison of selected morphological characters for species of *Neoniphon*. Data for *N. argenteus*, *N. aurolineatus* (as *Flammeo scythrops*), *N. sammara*, and *N. opercularis* are from Shimizu and Yamakawa (1979); data for *N. marianus* are from Woods (1955). Characters that differ from *N. pencei* are shown in bold. 1 as a proportion of Standard Length; 2 as a a proportion of orbit diameter; 3 as a proportion of head length.

Character	*N. pencei*	*N. argenteus*	*N. **aurolineatus***	*N. marianus*	*N. opercularis*	*N. sammara*
**Head Length^1^**	2.6-2.9	2.7-3.4	2.8-3.1	2.6-2.9	**2.9-3.1**	2.9-3.2
**Snout Length^2^**	1.2-1.4^2^ | 9.2-10.6^1^	1.2-1.6	1.2-1.5	9.5-10.6^1^	**0.8-1.0**	1.1-1.3
**Orbit Diameter^3^**	2.6-3.0^3^ | 7.4-8.5^1^	2.4-3.0	2.5-2.9	6.5-8.2^1^	**3.0-3.5**	2.5-3.0
**Interorbital Width^2^**	1.7-1.9^2^ | 12.5-15.4^1^	**1.2-1.7**	1.6-2.1	11.5-14.8^1^	**1.2-1.5**	**1.3-1.6**
**Upper-jaw Length^3^**	2.3-2.6^3^ | 6.3-7.0^1^	**2.2-2.3**	**2.0-2.3**	5.9-6.7^1^	**2.1-2.2**	2.2-2.4
**First Dorsal-spine Length^3^**	3.3-4.1^3^ | 9.1-12.1^1^	**2.4-3.1**	3.2-4.5	14^1^	3.1.-3.6	**2.2-3.0**
**Third Anal-spine Length^3^**	1.1-1.3^3^ | 3.1-3.8^1^	**1.4-1.6**	**1.4-1.6**	3.4-4.4^1^	**1.5-1.9**	1.1-1.5
**Fourth Anal-spine Length^3^**	1.7-2.0	**1.9-2.4**	**2.1-2.7**	-	**2.0-2.7**	1.8-2.2
**Dorsal-fin soft rays**	13	11-13	12-13	12-13	13	**11-12**
**Pectoral-fin Rays**	14	**12-13**	14	14	13-14	13-14
**Lateral-line Scales**	48-52	**38-43**	**44-46**	**46-47**	**38-40**	**39-43**
**Scales Above Lateral Line**	5	**2.5**	**3.5**	**3.5**	**2.5**	**2.5**
**Scales Below Lateral Line**	6-7	**7-8**	**8-9**	**8**	**8**	**8**
**Gill Rakers**	19-20	12-19	**15-17**	18-19	17-19	13-20
